# Absence of 2009 Pandemic H1N1 Influenza A Virus in Fresh Pork

**DOI:** 10.1371/journal.pone.0008367

**Published:** 2009-12-18

**Authors:** Amy L. Vincent, Kelly M. Lager, Michelle Harland, Alessio Lorusso, Eraldo Zanella, Janice R. Ciacci-Zanella, Marcus E. Kehrli, Alexander Klimov

**Affiliations:** 1 Virus and Prion Diseases Research Unit, National Animal Disease Center, United States Department of Agriculture, Agricultural Research Service, Ames, Iowa, United States of America; 2 Universidade de Passo Fundo, Campus 1, Bairro São José, Passo Fundo, Rio Grande do Sul, Brazil; 3 Labex-USA, EMBRAPA–Brazilian Agriculture Research Corporation, Brasilia, Distrito Federal, Brazil; 4 Influenza Division, Centers for Disease Control and Prevention, Atlanta, Georgia, United States of America; Tsinghua University, China

## Abstract

The emergence of the pandemic 2009 H1N1 influenza A virus in humans and subsequent discovery that it was of swine influenza virus lineages raised concern over the safety of pork. Pigs experimentally infected with pandemic 2009 H1N1 influenza A virus developed respiratory disease; however, there was no evidence for systemic disease to suggest that pork from pigs infected with H1N1 influenza would contain infectious virus. These findings support the WHO recommendation that pork harvested from pandemic influenza A H1N1 infected swine is safe to consume when following standard meat hygiene practices.

## Introduction

The emergence of the pandemic H1N1 2009 influenza A virus in humans and subsequent discovery that it was of swine influenza virus lineages [1] raised many questions about this novel virus. One such concern relates to food safety, if swine were to become infected with the pandemic virus would the meat be contaminated with virus and be a potential source of human infection? To address this question we tested non-respiratory tract tissues for virus following infection of young pigs with the pandemic H1N1 2009 virus.

## Materials and Methods

A total of 30 pigs were inoculated with A/CA/04/2009 (H1N1)v (n = 15 pigs) or A/Mexico/4108/2009 (H1N1)v (n = 15 pigs) as part of ongoing studies to determine the susceptibility of swine to the human virus (Vincent, unpublished). The animals were housed according to the National Animal Disease Center Institutional Animal Care and Use Committee guidelines. Five pigs from each virus challenge group were euthanized on 3, 5, and 7 days post infection (dpi). All pigs were treated similarly receiving an intratracheal challenge with 2×10^5^ 50% tissue culture infectious dose (TCID_50_) as previously described [2]. Postmortem samples including serum, lung, tonsil, liver, kidney, spleen, inguinal lymph node, colon contents (feces), and skeletal muscle from the *semitendinosus* were collected at necropsy using individual sterile instruments between tissues and between pigs. Non-challenged age-matched negative control pigs were necropsied at 7 dpi (n = 5 pigs).

The tissues were tested for virus by a real-time RT-PCR (qRT-PCR) specific for the pandemic H1N1 matrix gene (Lorusso, submitted) and virus isolation on Madin Darby Canine Kidney (MDCK) cells. Briefly, approximately 500 mg of tissue was homogenized in sterile phosphate buffered saline (PBS) with antibiotics using a power homogenizer with sterile generators at 20% w/v. The MagMax Microarray (Ambion) protocol for RNA extraction from tissues was followed using 100 µL of tissue homogenate. The MagMax Viral RNA Isolation (Ambion) kit protocol was used as per manufacturer's instructions for serum by adding 50 µL to the MagMax plate for RNA extraction. Viral RNA samples were tested in duplicate by qRT-PCR.

For virus isolation, 200 µL of the tissue homogenate or serum sample was placed on confluent MDCK cells in 24-well plates to incubate for 1 hr. After 1 hr of incubation the sample was removed and 400 µL MEM w/TPCK trypsin was added. The plate was checked at 24 and 48 hrs for cytopathic effects. After 48 hrs, 200 µL of cell culture supernatant from each well of the 24-well plate after one freeze and thaw cycle was subsequently passed onto a confluent 48 well plate. After 48 hrs, evidence of cytopathic effects was evaluated and presence of virus antigen confirmed by immuno-cytochemical staining. Virus titers in virus isolation positive tissue homogenates were determined on MDCK cells in 96-well plates.

## Results

Influenza virus was isolated from the lung tissue of all pigs euthanized on 3 and 5 dpi, and from the tonsil tissue of 1 pig in each virus challenge group (Table 1). The mean virus titers for lung tissue homogenates from pigs infected with CA/09 were 10^4.0^ and 10^2.8^ TCID_50_ per mL for days 3 and 5 pi, respectively. The mean virus titers for lung tissue homogenates from pigs infected with MX/09 were 10^3.9^ and 10^3.1^ TCID_50_ per mL for days 3 and 5 pi, respectively. The tonsil samples were below sensitivity limits of titration. The positive lung tissue was consistent with virus isolation from broncho-alveolar lavage fluid (BALF) (data not shown). All pigs were positive in BALF by virus isolation on days 3 and 5 pi, but negative on 7 dpi as demonstrated in the lung tissue. Influenza viral nucleic acid was detected by qRT-PCR in all of the 20 virus-positive lung tissues but from neither of the virus isolation positive tonsil samples (Table 2). In addition, viral nucleic acid was detected in the lung samples of all pigs at 7 dpi as well as one lymph node sample at 3 dpi, but these samples were virus isolation negative. The mean copy numbers by qRT-PCR for lung tissue homogenates from pigs infected with CA/09 were 10^4.3^, 10^4.7^, and 10^3.3^ for days 3, 5, and 7 pi, respectively. The mean copy numbers by qRT-PCR for lungs infected with MX/09 were 10^4.9^, 10^3.9^, and 10^2.7^ for days 3, 5, and 7 pi, respectively. The copy number of the positive lymph node sample was 10^2.4^. No infectious virus or viral nucleic acid was detected in any of the remaining tissue samples from any of the virus challenge pigs or from any of the negative control pigs. qRT-PCR was more sensitive for viral RNA in the lungs at 7 dpi, at which time the pigs were recovering clinically and viral shedding was declining. Importantly, qRT-PCR did not detect viral RNA in any internal organs or muscle tissue samples.

**Table 1:** Presence of pandemic H1N1 influenza virus by virus isolation in samples collected at 3, 5, and 7 days post infection (dpi).*

Day	Virus	Lung	Tonsil	LN	Serum	Spleen	Liver	Kidney	Feces	Muscle
3 dpi	CA/09	5	0	0	0	0	0	0	0	0
	MX/09	5	1	0	0	0	0	0	0	0
5 dpi	CA/09	5	1	0	0	0	0	0	0	0
	MX/09	5	0	0	0	0	0	0	0	0
7 dpi	CA/09	0	0	0	0	0	0	0	0	0
	MX/09	0	0	0	0	0	0	0	0	0

* 15 pigs were infected with either the A/CA/04/2009 (CA/09) or A/Mexico/4108/2009 (MX/09) pandemic H1N1 virus isolates. Number of pigs positive out of 5 is reported from each group euthanized on 3, 5, or 7 dpi; LN = inguinal lymph node tissue sample.

**Table 2:** Presence of pandemic H1N1 influenza virus by qRT-PCR in samples collected at 3, 5, and 7 days post infection (dpi).*

Day	Virus	Lung	Tonsil	LN	Serum	Spleen	Liver	Kidney	Feces	Muscle
3 dpi	CA/09	5	0	0	0	0	0	0	0	0
	MX/09	5	0	1	0	0	0	0	0	0
5 dpi	CA/09	5	0	0	0	0	0	0	0	0
	MX/09	5	0	0	0	0	0	0	0	0
7 dpi	CA/09	5	0	0	0	0	0	0	0	0
	MX/09	5	0	0	0	0	0	0	0	0

* 15 pigs were infected with either the A/CA/04/2009 (CA/09) or A/Mexico/4108/2009 (MX/09) pandemic H1N1 virus isolates. Number of pigs positive out of 5 is reported from each group euthanized on 3, 5, or 7 dpi; LN = inguinal lymph node tissue sample.

Clinical disease was induced in all infected pigs and will be reported elsewhere in detail (Vincent, unpublished). Infectious virus was detected in lungs from all experimentally infected pigs necropsied on 3 and 5 dpi, confirming infection for both CA/09 and MX/09. These observations are consistent with what has been reported with German and British experiments in which clinical disease was induced and infectious virus and viral nucleic acid could be detected in tissue samples associated with the respiratory tract [3], [4]. Neither infectious virus nor viral nucleic acid was detected in plasma samples collected on days −1 through 7 dpi [4]. Except for plasma collected from pigs in the British experiment, no other non-respiratory tract tissues were reported to have been tested for virus.

## Discussion

Experimental infections of swine with the pandemic H1N1 virus have described a clinical disease where pigs develop pyrexia, anorexia, and dyspnea within several days following challenge [3], [4] that is similar to what has been reported in endemic swine influenza virus experiments. Likewise, there have been reports of swine becoming infected in the field with the pandemic H1N1 virus in which the pigs displayed mild respiratory disease (http://www.oie.int/eng/en_index.htm). In these cases it is believed that the pigs became infected following contact with infected people. Collectively, this data suggests the pandemic H1N1 virus replicates in swine and produces clinical illness that is indistinguishable from typical swine influenza virus.

In contrast to highly pathogenic avian influenza virus infections in poultry [5], existing evidence suggests that swine influenza virus does not induce a systemic infection contaminating the meat, although there is limited data to support this assumption. To the authors' knowledge there are only two reports that describe an infrequent viremia in pigs during the acute phase of the infection with swine influenza virus [6], [7]. One of these papers also describes sporadic isolation of influenza virus from “other tissues such as intestine and muscle and from faeces,” however, the methodology is not well described and it is unclear during the acute infection which tissues were positive at which times [7]. PCR was not utilized in these studies and it is unknown if tissues would have been positive by this method. Infection of swine with highly pathogenic avian influenza virus induced minimal disease with no evidence for a systemic infection based on virus isolation and PCR [8]. Similarly, infection of swine with the 1918 Spanish flu virus resulted in minimal pulmonary disease with no virus being isolated from a variety of non-respiratory tissues [9].

In this study, tissues outside the respiratory tract were found to be negative by virus isolation on days 3, 5 and 7 pi for both isolates of 2009 pandemic H1N1 evaluated. Only lung and tonsil samples from days 3 and 5 pi were positive by virus isolation. In addition, 7 dpi lung samples and inguinal lymph node from one pig were positive for viral RNA, but were negative by virus isolation. This may be due to the increased sensitivity of the qRT-PCR in detecting viral RNA over the sensitivity of detecting viable virus by tissue culture techniques. By 7 dpi, viable virus is typically cleared from the lung in pigs with uncomplicated infection with influenza A virus, including 2009 pandemic H1N1 (Vincent, unpublished), thus the viral RNA is likely remains following activation of the host innate immune response. Two virus isolation positive tonsil samples were found to be negative by qRT-PCR. This is likely due to the low quantity of viable virus and/or viral RNA being at the threshold of sensitivity for both assays.

In summary, the 2009 pandemic H1N1 virus can induce respiratory disease in swine that is consistent with influenza illness. However, there was no evidence for systemic infection that would contaminate meat with infectious virus. It is important to note that ill swine would not be allowed entry into the U.S. food supply as per USDA Food Safety and Inspection Service criteria. However, the findings reported in this study support the WHO recommendation that pork harvested from 2009 pandemic influenza A H1N1 infected swine would be safe to consume when following standard meat hygiene practices (http://www.who.int/mediacentre/news/statements/2009/h1n1_20090430/en/index.html).
